# Multi-Component Botanical Crude Extracts Improve Egg and Meat Quality in Late-Laying Hens Through Gut Microbiota Modulation

**DOI:** 10.3390/foods14203480

**Published:** 2025-10-12

**Authors:** Xiaofang Wei, Huixin Liu, Fang Chen, Yumiao Liang, Wenwen Yang, Wenjing Liang, Ting Xu, Hongjie Hu, Xiuyu Li, Hongbin Si, Shuibao Shen

**Affiliations:** 1 College of Animal Science and Technology, Guangxi University, Nanning 530004, China; xiaofangwei0504@163.com (X.W.); 18345760639@163.com (T.X.);; 2 Guangxi Zhuang Autonomous Region Veterinary Drug Inspection Institute, Nanning 530004, China; 3 Agricultural and Livestock Industry Development Research Institute, Guangxi University, Nanning 530004, China

**Keywords:** gut microbiota, serum metabolomics, egg quality, meat quality, laying hens, botanical extracts

## Abstract

Laying hens in the late laying period often experience reduced productivity and declining egg and meat quality, which limits breeding efficiency and resource utilization. This study aimed to evaluate the effects of multi-component Botanical Crude Extracts (BCEs) on egg and meat quality, metabolic health, and gut microbiota in aged laying hens. A total of 4320 hens were supplemented with 0.3% BCEs for 100 days, with evaluations at 60 and 100 days. BCE supplementation significantly enhanced egg flavor by promoting aromatic and fat-soluble volatiles and reducing odorous compounds (*p* < 0.05). BCEs improved yolk nutrition by enriching n-3 polyunsaturated fatty acids, especially docosahexaenoic acid (DHA), and optimizing the n-6/n-3 ratio (*p* < 0.05). A moderate reduction in amino acids was observed, which may reduce bitterness and ammonia burden (0.05 ≤ *p* < 0.10, trend). In muscle, BCEs improved protein–fat distribution, increased intramuscular fat, and enhanced flavor-related metabolites, significantly improving meat quality of culled hens (*p* < 0.05). BCEs also reshaped gut microbiota, reducing harmful taxa and promoting short-chain fatty acid and aromatic metabolite biosynthesis (*p* < 0.05). Serum metabolomics revealed modulation of AMPK, calcium, and cholesterol pathways, improving antioxidant capacity and lipid regulation (*p* < 0.05). Correlation analyses linked beneficial bacteria and metabolites with yolk DHA levels and flavor (*p* < 0.05). Overall, BCEs enhanced egg and meat quality and physiological health, providing guidance for functional feed strategies in aged laying hens.

## 1. Introduction

As the breeding cycle of egg-laying chickens is extended, they often experience problems such as decreased egg-laying performance and deteriorating egg and muscle quality in the later stages. This affects breeding benefits and restricts the effective utilization of resources from eliminated egg-laying chickens [1]. In recent years, the improvement of the quality of poultry products while ensuring their health has become a major focus of research in the domain of poultry nutrition and functional feed. Research has demonstrated that intestinal flora fulfills a pivotal function in the nutritional digestion and immune homeostasis regulation of animals. Furthermore, it has been established that intestinal flora plays a crucial role in the metabolism of fatty acids, the conversion of amino acids, and the production of volatile flavor substances [2]. However, as the egg-laying cycle progresses, the intestinal microecology of laying hens often shows changes such as reduced diversity, fewer beneficial bacteria, and metabolic dysfunction. These changes become an important cause of reduced nutrient conversion efficiency and deterioration of egg and meat quality [1,3]. BCEs have seen a surge in popularity in recent years as a means of regulating the intestinal flora-metabolite axis, thereby enhancing animal health and product quality. This is due to the natural, safe, and wide-ranging targets of BCEs [4]. BCEs are rich in polysaccharides [5], flavonoids [6], chlorogenic acid [7], and other active substances, and have good antioxidant, anti-inflammatory, and intestinal flora regulation potential. However, systematic research is lacking to reveal the specific pathways by which this “microbiome-metabolite” linkage improves egg and meat quality in late-laying hens. In particular, how the microbiome and its metabolites regulate flavor and nutritional quality in late-laying hens remains a research gap.

**Figure 1:**
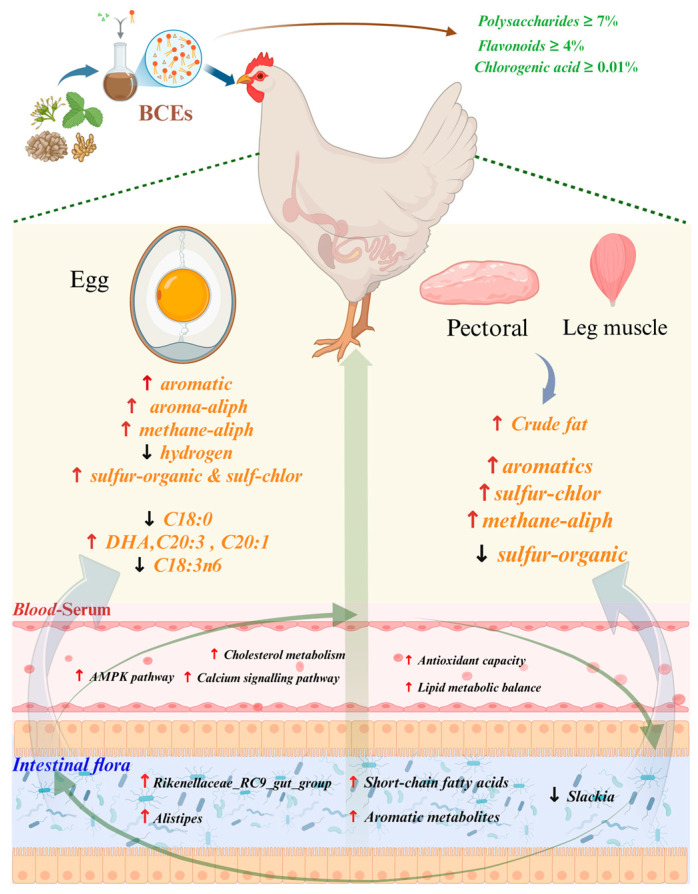
BCEs modulate gut microbiota-metabolite axis to improve egg and meat quality in late-laying hens. Red upward-pointing arrows represent an increase, whereas black downward-pointing arrows represent a decrease.

We hypothesized that BCEs, through their multi-component bioactive profile, would systemically improve egg and meat quality by remodeling the gut microbiota and subsequently modulating host metabolic pathways related to lipid, protein, and flavor compound metabolism. Based on this, this study, focusing on two key time points, 60 and 100 days of age, systematically analyzed the mechanisms by which BCEs influence the “microbiome-metabolite-flavor/nutrient” regulatory axis in late-laying hens. This study aims to provide a theoretical basis and data support for improving the quality of laying hen products and developing functional feeds. The complete research process is illustrated in Figure 1.

## 2. Materials and Methods

### 2.1. Ethics Statement

All experiments involving animals were performed in accordance with the ethical policies and procedures approved by the Animal Ethics Committee of Guangxi University (GXU-2023-0185).

### 2.2. Experimental Animals and Feeding Management

This study enrolled 4320 670-day-old Dawu Jinfeng late-laying hens in good health and of consistent weight. A randomized design was used with two treatment groups: a CON (basal diet) and a BCEs (basal diet supplemented with 0.3% BCEs) group, with 2160 birds in each group and six replicates per group, each with 360 birds per replicate. The natural herbal formula used in the study consisted of crude extracts from natural plants, including honeysuckle, mulberry leaves, Codonopsis pilosula, and Magnolia officinalis, homogenized to form a stable aqueous solution. The main active ingredients in the formula (based on the weight of the stock solution) were as follows: polysaccharides ≥ 7%, flavonoids ≥ 4%, chlorogenic acid ≥ 0.01%, and neochlorogenic acid ≥ 0.002%, cryptochlorogenic acid ≥ 0.003%. The BCE dosage was calculated at 0.3% of feed intake daily, accurately weighed, dissolved in the drinking water throughout the day, thoroughly stirred, and provided continuously 24 h a day via an automatic nipple drinking system. The control group received plain water without additives. Aside from the additives, the two groups maintained the same housing environment, management methods, and basal diet. The experimental period lasted 100 days, divided into two phases: the initial phase (days 0–60) and the final phase (days 61–100). All birds were fed the same basal diet throughout the experimental period. The detailed composition and nutrient levels are detailed in Table 1.

### 2.3. Sample Collection and Processing

#### 2.3.1. Blood and Tissue Sampling

On days 60 and 100 of the experiment, two laying hens were randomly selected from each replicate in each group (a total of 2 groups × 6 replicates × 2 laying hens = 24 laying hens) for body weight measurement. Subsequently, approximately 4 mL of blood was collected from the sub-wing vein in a standard vacuum tube without an anticoagulant. The blood was tilted and allowed to stand for 30 min to allow for natural coagulation and serum precipitation. After separation, the blood was immediately stored at −80 °C until further use. After blood collection, the hens were euthanized and depilated. The dressing rate, evisceration rate, and evisceration rate were determined. Pectoral and leg muscle tissue, as well as cecal contents, were collected for analysis of meat quality, nutritional composition, and intestinal flora.

#### 2.3.2. Egg Collection

On days 60 and 100 of the experiment, two eggs laid that day (a total of 48 eggs) were randomly collected from each replicate in each group for egg quality analysis. Among them, one fresh whole egg and one steamed egg were collected from each replicate for flavor substance detection; another one fresh egg yolk liquid and one egg yolk and egg white mixture were taken for nutritional component and flavor index determination.

### 2.4. Measurement Indicators and Analysis Methods

#### 2.4.1. Electronic Nose Detection

One fresh egg was randomly selected from each replicate group, cracked open, placed in a clean container, and thoroughly blended using a blender to completely blend the egg white and yolk. This mixture was then prepared as an egg white and yolk mixture. Another egg was cooked in a poacher for 13 min, cooled to room temperature, peeled, quickly ground, and thoroughly blended to obtain a cooked egg sample. For meat samples, one piece of fresh pectoral and one piece of leg muscle tissue were each sampled from each replicate group and blended in a food processor for 30 s to prepare homogenized breast and leg muscle samples. Six grams of each sample (raw egg, cooked egg, pectoral, and leg muscle) were weighed and placed in a 15 mL sealed vial. The sample was then equilibrated at room temperature for 30 min. Five replicates were performed for each sample. Odor analysis was performed using an electronic nose analyzer (PEN3, Airsense Analytics GmbH, Schwerin, Germany) with the following instrument parameters: 180 s cleaning time, 10 s zeroing time, 5 s sample preparation time, 150 s detection time, 300 mL/min carrier gas flow rate, and 300 mL/min injection flow rate. The sensor types used and their corresponding sensitive gases are detailed in Supplementary Table S1.

#### 2.4.2. Amino Acid and Fatty Acid Profile Analysis

For each replicate, one fresh egg was separated, and the yolk was quickly stirred to prepare egg yolk liquid. Another fresh egg was cracked and placed in a container. The egg white and yolk were quickly stirred using a blender to prepare an egg yolk-egg white mixture. The fatty acid content of the egg yolk liquid and the egg yolk-egg white mixture was determined using a gas chromatograph (Agilent 7890-B, Santa Clara, CA, USA) according to method 1 of GB 5009.168-2016 [8]. The content of 16 amino acids was determined using a Hitachi amino acid analyzer (Hitachi 1915-002, Hitachinaka, Japan) according to GB 5009.124-2016 [9].

#### 2.4.3. Determination of Slaughter Performance, pH, Meat Colored, Drip Loss, Crude Protein, Crude Fat

After a 12 h fasting period, chickens were humanely slaughtered by external cervical exsanguination in accordance with the animal ethics guidelines (GXU-2023-0185). Pre-slaughter live weight (kg) was recorded, and the following parameters were measured: carcass weight: carcass weight after exsanguination, feather removal, and removal of leg cuticles, toe shells, and beak shells (kg); slaughter rate (%) = carcass weight/pre-slaughter live weight × 100%; semi-eviscerated weight: the weight (kg) of the carcass after dissection, retaining the liver, heart, gizzard, proventriculus, kidney, lungs, and abdominal fat; semi-eviscerated rate (%) = semi-eviscerated weight/live weight before slaughter × 100%; full eviscerated weight: the weight after removing the head and feet on the basis of semi-eviscerated, retaining only the kidneys (kg); eviscerated rate (%) = eviscerated weight/live weight before slaughter × 100%; abdominal fat weight: total weight including abdominal fat and fat around the gizzard (g); abdominal fat rate (%) = abdominal fat weight/(eviscerated weight + abdominal fat weight) × 100%; pectoral weight: weight of the breast muscle after skin and bones are removed (kg); pectoral rate (%) = bilateral breast muscle weight/eviscerated weight × 100%; leg muscle weight: weight of the leg muscle after skin, bones, and subcutaneous fat are removed (kg); leg muscle rate (%) = bilateral leg muscle weight/eviscerated weight × 100%; lean rate (%) = (breast muscle weight + leg muscle weight)/eviscerated weight × 100%. Muscle pH was measured using a calibrated portable pH meter (Testo 205, Testo GmbH, Titisee-Neustadt, Germany). The instrument was calibrated with standard pH buffers (e.g., pH 4.00, 7.00) prior to use. Measurements were taken in triplicate at different locations within the pectoral and leg muscles, and the average value was calculated. Instrumental meat color assessment was performed using a chroma meter (OPTO-LAB CR-410, Konica Minolta, Tokyo, Japan). The instrument was configured with a D65 light source, a 10° standard observer angle, and an enclosed measuring cone to exclude ambient light. The L* (lightness), a* (redness), and b* (yellowness) values were recorded from at least three random locations on both the pectoral and leg muscles, and the mean values were used for subsequent analysis. Based on the averaged a* and b* values, the chromaticity parameters were calculated as Chroma (saturation) as (a^2^ + b^2^)^0.5 and Hue Angle as arctan (b*/a*). Meat color was measured using an OPTO-LAB (CR-410, Konica Minolta, Japan) meat colorimeter, with measurements taken at least three times at different locations in the pectoral and leg muscles, and the average value was calculated. Results are expressed as L* (lightness), a* (redness), and b* (yellowness).

To determine the drip loss rate of pectoral and leg muscles, equal volumes of pectoral muscle (approximately 5 cm long, 3 cm wide, and 2 cm thick) were collected from the same side along the muscle fiber direction. The initial mass, W1, was measured. The sample was suspended with a silk thread in a sealed container at 4 °C for 24 h. After 24 h, the sample was removed, the surface moisture was blotted dry, and the mass, W2, was measured. Three replicates were set for each sample, and the 24 h drip loss rate was calculated: 24 h drip loss rate = [(W1 − W2)/W1] × 100%. For nutrient composition determination, crude protein content was determined using an automated Kjeldahl nitrogen analyzer according to the national standard GB/T 6432-2018 [10]; crude fat content was determined using Soxhlet extraction according to GB/T 6433-2006 [11].

#### 2.4.4. 16S rRNA Sequencing and Bacterial Diversity Analysis

Total genomic DNA from the 100-day-old cecal microbial community was extracted using the E.Z.N.A.^®^ soil DNA kit (Omega Bio-tek, Norcross, GA, USA). Quality and purity were assessed by 1% agarose gel electrophoresis and NanoDrop 2000 (Thermo Scientific, Waltham, MA, USA). The 16S rRNA gene V3-V4 region was amplified using primers 338F/806R (20 μL system: 10 ng template DNA, 27 cycles). PCR products were purified by 2% gel purification and quantified using Qubit 4.0 (Thermo Fisher Scientific, USA). Libraries were prepared using the NEXTFLEX Rapid DNA-Seq Kit (Bioo Scientific, Austin, TX, USA), and sequenced on an Illumina Nextseq 2000 (Illumina, Inc., San Diego, CA, USA). The bioinformatics analysis process included fastp quality control (cutting off bases with a quality value < 20 and filtering low-quality and N-reads); FLASH assembly (minimum overlap 10 bp, mismatch rate ≤ 0.2); UPARSE clustering of OTUs at 97% similarity and removal of chimeras; excluding chloroplast/mitochondrial sequences and averaging to 20,000 reads per sample (Good’s coverage reached 99.09%); RDP classifier alignment of species annotations to the Silva (v138) database (confidence level 70%); and the PICRUSt2 prediction function. All data analyses were performed on the MajorBio cloud platform (https://cloud.majorbio.com): calculation of the Chao1/Shannon index (Wilcoxon test for intergroup differences); Bray–Curtis PCoA + PERMANOVA analysis of community structure differences; LEfSe screening of differentially expressed groups (LDA > 2, *p* < 0.05); db-RDA and linear regression analysis of the association between environmental factors and communities; and construction of a co-occurrence network based on Spearman correlation (|r| > 0.6, *p* < 0.05).

#### 2.4.5. Non-Targeted Metabolomics Analysis

The main steps of serum non-targeted metabolome analysis are sample preparation (100-day serum samples), LC-MS/MS analysis, data preprocessing and database search, and differential metabolite analysis. The specific steps are as follows: (1) Sample preparation and extraction: The sample stored in a refrigerator at −80 °C was thawed on ice and vortexed for 10 s. Then, 50 μL of sample and 300 μL of extraction solution (ACN: Methanol = 1:4, *V*/*V*) containing internal standards were added into a 2 mL microcentrifuge tube. The sample was vortexed for 3 min and then centrifuged at 12,000 rpm for 10 min (4 °C). Then, 200 μL of the supernatant was collected and placed in −20 °C for 30 min, and then centrifuged at 12,000 rpm for 3 min (4 °C). Then, 180 μL aliquots of supernatant were transferred for LC-MS analysis. (2) HPLC Conditions: all samples were for two LC/MS methods. One aliquot was analyzed using positive ion conditions and was eluted from T3 column (Waters ACQUITY Premier HSS T3 Column 1.8 µm, 2.1 mm × 100 mm) using 0.1% formic acid in water as solvent A and 0.1% formic acid in acetonitrile as solvent B in the following gradient: 5 to 20% in 2 min, increased to 60% in the following 3 min, increased to 99% in 1 min and held for 1.5 min, then come back to 5% mobile phase B within 0.1 min, held for 2.4 min. The analytical conditions were as follows: column temperature, 40 °C; flow rate, 0.4 mL/min; injection volume, 4 μL; Another aliquot was used under negative ion conditions and was the same as the elution gradient of positive mode. (3) MS Conditions (AB): The data acquisition was operated using the information-dependent acquisition (IDA) mode using Analyst TF 1.7.1 Software (Sciex, Concord, ON, Canada). The source parameters were set as follows: ion source gas 1 (GAS1), 50 psi; ion source gas 2 (GAS2), 50 psi; curtain gas (CUR), 25 psi; temperature (TEM), 550 °C; declustering potential (DP), 60 V, or −60 V in positive or negative modes, respectively; and ion spray voltage floating (ISVF), 5000 V or −4000 V in positive or negative modes, respectively. The TOF MS scan parameters were set as follows: mass range, 50–1000 Da; accumulation time, 200 ms; and dynamic background subtraction. The product ion scan parameters were set as follows: mass range, 25–1000 Da; accumulation time, 40 ms; collision energy, 30 or −30 V in positive or negative modes, respectively; collision energy spread, 15; resolution, UNIT; charge state, 1 to 1; intensity, 100 cps; exclude isotopes within 4 Da; mass tolerance, 50 ppm; maximum number of candidate ions to monitor per cycle, 18. The original data file acquired by LC-MS was converted into mzXML format by ProteoWizard software (version 3.0.21076; http://proteowizard.sourceforge.net/). Peak extraction, peak alignment, and retention time correction were, respectively, performed by the XCMS program. The “SVR” method was used to correct the peak area. The peaks with a detection rate lower than 50% in each group of samples were discarded. After that, metabolic identification information was obtained by searching the laboratory’s self-built database (metware database), integrated public database, AI database, and metDNA.

### 2.5. Statistical Analysis

Data were processed using Microsoft Excel 2020 and statistically analyzed using SPSS 27.0 (IBM Corp., Armonk, NY, USA). Between-group comparisons were performed using an independent samples t-test. All data are presented as mean ± standard error of the mean (SEM). Statistical significance was defined as *p* < 0.05, while 0.05 ≤ *p* < 0.10 was considered indicative of a statistical trend.

Correlation analyses were conducted using Pearson correlation coefficients (r). The strength of correlations was interpreted as follows: |r| ≥ 0.8 represented very strong correlation, 0.6 ≤ |r| < 0.8 represented strong correlation, 0.4 ≤ |r| < 0.6 represented moderate correlation, and |r| < 0.4 represented weak correlation. All correlation coefficients were tested for statistical significance, with *p* < 0.05 considered statistically significant.

## 3. Results

### 3.1. Effects of Feeding BCEs on Egg Quality and Flavor

To systematically evaluate the regulatory effects of BCEs on egg quality during the late laying period, this study analyzed egg flavor characteristics, fatty acid composition, and amino acid content. Regarding flavor characteristics, electronic nose detection results (Table 2 and Table 3) showed that BCEs significantly modulated volatile odor components in eggs, with this effect exhibiting a time-dependent and sensor-specific effect. At 60 days of age, the response values of aroma-related sensors (aromatic, broadrange, and aroma-aliph) in raw eggs were significantly enhanced (*p* < 0.05), and methane-aliph also increased significantly (*p* = 0.001), indicating an increase in the content of aromatic and fat-soluble flavor compounds. The response to hydrogen was significantly decreased (*p* < 0.01), indicating a decrease in the release of odorous gases. The response to sulfur-organic and sulf-chlor sensors was enhanced (*p* < 0.05), potentially contributing to the enhanced yolk flavor. The sensor changes in cooked eggs were consistent with those in raw eggs, with even more significant differences (*p* < 0.01), further validating the flavor-enhancing effect of BCEs. By 100 days of age, the responses of the aromatic, aroma-aliph, and methane-aliph sensors in raw eggs were further enhanced (*p* < 0.05), while the responses to hydrogen and broad-methane were significantly reduced (*p* < 0.05). The responses to aromatic, sulfur-chlor, and methane-aliph sensors in cooked eggs were significantly higher than those in the control group (*p* < 0.01). These results suggest that BCEs can enhance the richness and harmony of egg flavor by promoting the accumulation of aromatic, aliphatic, and sulfur-containing volatiles, and this effect is more pronounced during the later stages of egg production.

Fatty acid composition analysis (Table 4 and Table 5) indicated that BCEs contribute to the optimization of egg yolk lipid structure. At 60 days of age, BCEs significantly reduced the saturated fatty acid C18:0 content (*p* = 0.043), while significantly increasing C20:3 (*p* = 0.036) and C22:6 (DHA, *p* = 0.045), improving the ratio of n-6 to n-3 polyunsaturated fatty acids. Furthermore, C20:1 content increased significantly (*p* = 0.044), while C18:3n6 decreased significantly (*p* = 0.043), suggesting that BCEs may promote fatty acid elongation and conversion. At 100 days of age, DHA content increased further (*p* = 0.031), while C18:3n6 and C20:3 contents decreased (*p* = 0.033 and 0.040), respectively. The upward trend in C20:1 did not reach significance (*p* = 0.083). Overall, BCEs promoted the deposition of high-nutritional-value fatty acids without increasing saturated fatty acids, thereby improving the nutritional structure of egg yolk lipids.

Amino acid composition analysis (Table 6 and Table 7) showed that BCEs had a certain inhibitory effect on the amino acid content of the egg yolk and egg white mixture. At 60 days of age, the contents of glycine, methionine, tyrosine, and histidine were significantly reduced (*p* < 0.05), while alanine, isoleucine, and proline showed a decreasing trend (0.05 < *p* < 0.10). The total content of 16 amino acids also showed a decreasing trend (*p* = 0.08). At 100 days of age, the contents of glycine and methionine continued to decrease significantly (*p* < 0.05), while the downward trend of histidine and proline continued. The contents of several amino acids, including threonine, lysine, and phenylalanine, also showed a decreasing trend (0.05 < *p* < 0.10). The total content of 16 amino acids further decreased (*p* = 0.11). These results suggest that BCEs may inhibit protein synthesis or amino acid transport to some extent.

In summary, 0.3% BCEs can effectively enhance egg flavor characteristics and optimize fatty acid composition during the late egg-laying period, while also inhibiting amino acid accumulation to a certain extent. This combined effect enhances the deposition of aromatic and functional lipids, reduces potential off-flavor release, and reduces amino acid excess, potentially offering application value in improving the nutritional and sensory quality of eggs.

### 3.2. Feeding BCEs Improves the Chicken Meat Quality and Flavor of Laying Hens in the Late Laying Period

Regarding slaughter performance (Table 8), the BCE group showed a significantly higher dressing percentage than the control group at 60 days of age (*p* = 0.011). Although liveweight, carcass weight, semi-eviscerated weight, full-eviscerated weight, and abdominal fat weight all showed increasing trends, the differences did not reach significance (*p* > 0.05). Breast and leg muscle weights, as well as their related tissue proportions (pectoral rate, leg muscle rate, abdominal fat rate, and lean meat rate), did not differ significantly between the two groups (*p* > 0.05). By 100 days of age, all slaughter parameters remained unchanged between the two groups, although the BCE group maintained higher values for these parameters overall. Analysis of the interaction effect of time and treatment revealed that the improvement in dressing percentage with BCEs occurred primarily at 60 days of age (*p* < 0.05), suggesting a windowed effect, likely through promoting abdominal cavity development and fat deposition (as evidenced by the increasing trends in abdominal fat and eviscerated weight), while having relatively limited direct effects on muscle tissue.

Regarding meat quality traits and nutritional composition (Table 9, Table 10 and Table 11), BCEs had no significant effects on pH, meat color parameters (L*, a*, b*), or drip loss in breast and leg muscles at either 60 or 100 days of age (*p* > 0.05), indicating limited regulatory effects on muscle color and water retention. Regarding nutritional composition, crude protein and crude fat content did not differ significantly between the two groups at 60 days of age (*p* > 0.05). However, by 100 days of age, crude protein content in pectoral significantly decreased (*p* = 0.034) and crude fat content significantly increased (*p* = 0.044) in the BCEs group. Leg muscle also showed a trend of decreased crude protein (*p* = 0.060) and increased crude fat (*p* = 0.051), suggesting that long-term BCEs treatment may induce a redistribution of muscle protein and lipid metabolism, significantly altering muscle nutritional composition.

Regarding flavor characteristics (Table 12), electronic nose analysis revealed that BCEs significantly modulated the volatile composition of chicken meat, demonstrating distinct sensor response specificity and time dependence. At 60 days of age, the BCEs group exhibited significantly enhanced sensor responses for aromatics (other classification), sulfur-chlors, broad-methanes, alcohols/aldehydes, long-chain alkanes (methane-aliphs), and aliphatic sulfides (sulfur-aliphs) (*p* < 0.05), while exhibiting significantly decreased sensor responses for aromatic compounds, nitrogen oxides, and sulfur-organics (*p* < 0.05). This suggests that BCEs may improve muscle aroma characteristics by modulating key metabolic pathways for aromatics, sulfur-containing compounds, and aldehydes. By 100 days of age, differences in responses to hydrogen, sulfate-aliph, alcohol/aldehyde, and methane-aliph persisted (*p* < 0.05), while the response directions of sulfate-chlor and sulfate-organic sensors reversed, and differences in aromatic and nitrogen oxide sensors disappeared (*p* > 0.05). In addition, broad-methane continued to increase in the BCEs group, but the difference did not reach a significant level (*p* = 0.072), suggesting that it may have a certain cumulative or hysteresis effect.

In summary, BCEs can improve slaughter performance, change muscle nutritional composition, and dynamically optimize the accumulation of volatile flavor compounds within a certain period of time by regulating lipid metabolism and aroma precursor pathways. They show strong time dependence and tissue specificity and have potential application value in improving the flavor and nutritional quality of laying hen products in the late egg-laying period.

### 3.3. Feeding BCEs Reshapes the Cecal Microbial Community Structure of Laying Hens in the Late Laying Period

**Figure 2:**
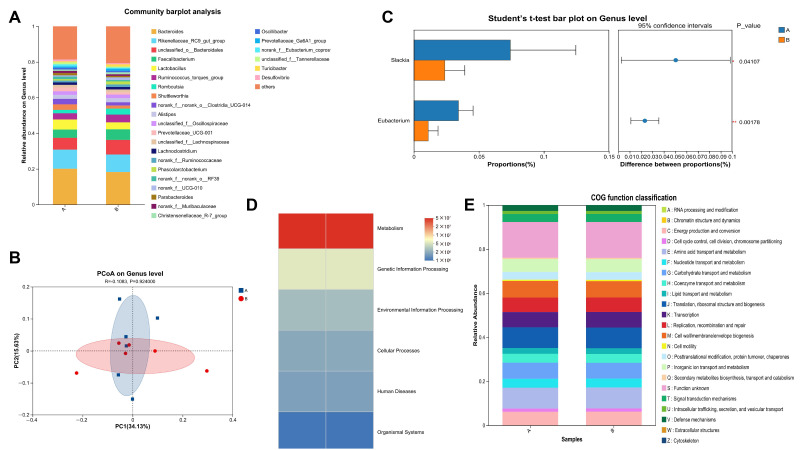
(**A**) Bar chart analysis of community composition (Genus levels), (**B**) PCoA analysis (Genus levels), (**C**): Genus-level species difference analysis, (**D**,**E**) PICRUSt2 function prediction. Group A = CON group, Group B = BCEs group.

Alpha diversity analysis (Figure S1) revealed no significant differences between the BCEs and CON groups in the Ace, Chao, Shannon, and Simpson indices (*p* > 0.05). However, the BCEs group showed an upward trend in the Ace and Chao indices, suggesting a potential role in enhancing the richness of the gut microbiota. Species composition and commonality analysis (Figure S2) revealed that at the OTU level, the two groups shared 1206 core OTUs, accounting for 45.02%. The CON and BCE groups had 394 unique OTUs (26.17%) and 393 unique OTUs (23.61%), respectively, for a total of 2162 and 2171 OTUs, suggesting that BCEs may modulate the composition and structure of the unique microbiota. OTU-level bar plots revealed some differences in the relative abundance of dominant OTUs between the two groups, reflecting their potential to regulate the fine structure of the microbiota. Further, at the phylum level, the relative abundances of major dominant phyla (such as *Firmicutes* and *Bacteroidetes*) were generally similar, but secondary phyla such as Actinobacteria and Desulfovibrionota showed a shift in their proportions. Genus-level bar plot analysis (Figure 2A) revealed significant differences in the dominant genus composition between the two groups. The relative abundance of some potentially probiotic genera (such as fiber-degrading bacteria or short-chain fatty acid-producing bacteria) increased in the BCEs group, suggesting that BCEs selectively enrich for core functional microbiota at the genus level. β-diversity analysis (Figure S3) revealed significant overlap in the PCoA distribution of OTUs between the two groups, with no significant differences between the groups (*p* > 0.05). Phylum-level PCoA analysis revealed a trend toward separation (*p* = 0.054). Furthermore, at the genus-level PCoA (Figure 2B), the two sample points were clearly separated (*p* < 0.05), indicating that BCEs had a more pronounced regulatory effect on the genus-level microbial community structure. Intergroup distance analysis further confirmed this trend: at both the phylum and genus levels, the Bray–Curtis distance values of the BCEs group were significantly higher than those of the control group (*p* < 0.01 and *p* < 0.05), indicating a significant remodeling of the overall intestinal microbiome structure. Genus-level difference analysis (Figure 2C) further revealed that the relative abundances of Slackia (*p* = 0.04107) and Eubacterium (*p* = 0.00178) were significantly lower in the BCEs group than in the control group, suggesting that BCEs may inhibit the overgrowth of certain potentially pathogenic or spoilage bacterial genera. Functional prediction analysis (Figure 2D,E) revealed an overall increase in the relative abundance of the “Metabolism” pathway in the BCEs group, as predicted by PICRUSt2, suggesting that BCEs may enhance the metabolic activity of the intestinal microbiome. COG functional annotation further showed that the BCEs group had a slightly increased relative abundance in the “synthesis, transport and degradation of secondary metabolites” pathway. This type of pathway usually involves the synthesis of functional metabolites such as short-chain fatty acids (SCFAs) and polyphenol derivatives. It may participate in the host’s energy metabolism and immune homeostasis regulation by promoting the production of beneficial metabolites, providing a functional basis for further clarifying the microecological intervention mechanism of BCEs.

### 3.4. Feeding BCEs Regulates Serum Metabolome Profile of Laying Hens in Late Laying Period

**Figure 3:**
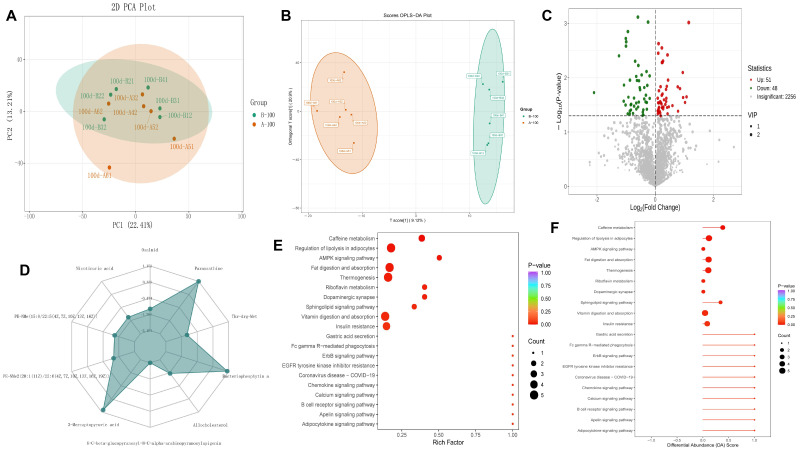
Non-targeted metabolomics analysis of serum after 100 days. (**A**): PCA, (**B**): OPLS-DA analysis, (**C**): Volcano plot of differential metabolites, (**D**): Radar plot of differential metabolites (top 10 metabolites with the largest absolute log_2_FC values), (**E**): KEGG enrichment plot of differential metabolites (top 20 pathways ranked by *p*-value, displayed in ascending order), (**F**): Differential abundance score plot (top 20 pathways ranked by *p*-value, displayed in ascending order). Group A = CON group, and Group B = BCE group.

The serum metabolite profile was significantly reshaped by BCEs treatment, as initially indicated by principal component analysis (PCA). The PCA score plot (Figure 3A) showed a clear separation trend, with PC1 explaining 22.41% of the total variance—a level of variance explanation considered biologically meaningful and typical for complex datasets in the field [12]. To statistically reinforce this observation and control for unrelated metabolic variance, a supervised orthogonal partial least squares-discriminant analysis (OPLS-DA) was employed. The OPLS-DA model (Figure 3B) yielded a definitive group separation, conclusively verifying the significant differences in metabolite composition between the BCEs and control groups. The score plot showed complete separation between the two groups, with a model goodness-of-fit (T-score) of 1.2%, indicating a robust and reliable model and a significant effect of BCEs on the systemic metabolite profile. Differential metabolite screening (Figure 3C) identified 99 significantly differentially expressed metabolites, using VIP > 1 and *p* < 0.05 as criteria. Of these, 51 were upregulated and 48 were downregulated in the BCEs group. Radar plot analysis (Figure 3D) revealed that the top 10 differentially expressed metabolites with the largest absolute log_2_ (fold change) values, including niacin, paraxanthine, and apigenin, all showed significant differences in expression between the two groups, suggesting that they may be key metabolic markers. KEGG enrichment analysis (Figure 3E) revealed that the differentially expressed metabolites were primarily enriched in functional pathways such as caffeine metabolism, AMPK signaling, ErbB signaling, chemokine signaling, and calcium signaling, implicated in key biological processes such as energy metabolism, cell signaling, and immune regulation. Caffeine metabolism and the AMPK signaling pathway were the most significantly enriched, with the lowest *p*-values. Further differential pathway abundance analysis (Figure 3F) revealed significant differences in the relative abundance of key metabolites in metabolic pathways such as adipose cytokine signaling, insulin resistance, and cholesterol metabolism between the two groups, suggesting that BCEs may regulate overall host metabolic homeostasis by affecting lipid, carbohydrate, and cholesterol metabolism. In summary, feeding BCEs significantly changed the serum metabolome profile of laying hens in the late laying period, affecting multiple metabolic pathways and their key metabolites, providing an important theoretical basis and target reference for further analysis of the metabolic regulation mechanism of BCEs.

### 3.5. Integrative Network Analysis of Microbiota, Metabolites and Quality Characteristics of Laying Hens in the Late Egg Production Period

**Figure 4:**
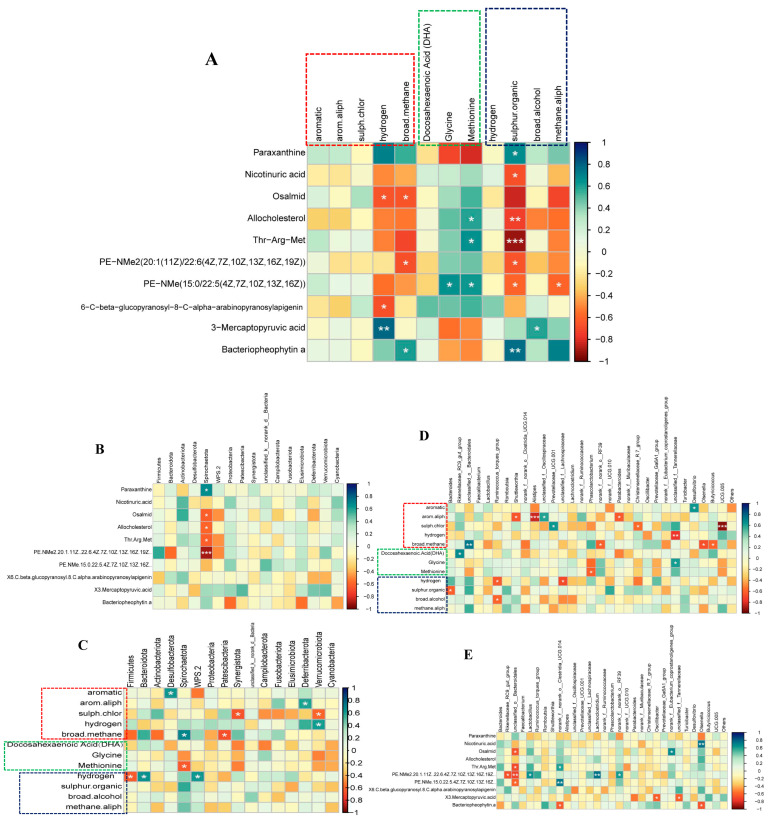
Correlation analysis between flavor, nutrition, metabolites, and microbial communities. (**A**): Correlation analysis between the egg electronic nose (red frame), egg amino acids and fatty acids (green frame), and leg muscle electronic nose (blue frame), and the top 10 differentially expressed metabolites with the largest absolute log_2_FC values in serum. (**B**): Correlation analysis between the top 10 differentially expressed metabolites with the largest absolute log_2_FC values in serum and phylum-level species abundance. (**C**): Correlation analysis between the egg electronic nose (red frame), egg amino acids and fatty acids (green frame), and leg muscle electronic nose (blue frame) and phylum-level species abundance. (**D**): Correlation analysis between the egg electronic nose (red frame), egg amino acids and fatty acids (green frame), and leg muscle electronic nose (blue frame) and genus-level species abundance. (**E**): Correlation analysis between the top 10 differentially expressed metabolites with the largest absolute log_2_FC values in serum and genus-level species abundance. * *p* < 0.05, ** *p* < 0.01, *** *p* < 0.001.

As shown in Figure 4, we conducted a systematic correlation analysis of the 10 metabolites with the highest absolute log_2_FC values in serum with electronic nose flavor profiles, nutritional components (amino acids and fatty acids), and microbial communities (at the phylum and genus levels), revealing their potential roles in regulating egg and muscle quality during late egg production. In the correlations between serum metabolites and phenotypic indicators (Figure 4A), paraxanthine showed a significant positive correlation with the “hydrogen” signature of the leg muscle electronic nose (r = 0.706), while showing a strong negative correlation with the amino acid glycine (r = −0.709), suggesting its potential involvement in regulating muscle energy metabolism and amino acid balance. Nicotinuric acid showed a moderate negative correlation with methionine (r = −0.582), suggesting a possible link between purine and sulfur amino acid metabolism. Bacteriopheophytin a was highly positively correlated with the “sulfur.organic” signature of the egg electronic nose (r = 0.762), suggesting a close association with the production of sulfur flavor precursors. Correlation analysis between serum metabolites and microbial phyla (Figure 4B) revealed that Bacteroidota showed a significant positive correlation with the “hydrogen” flavor signature (r = 0.641), while Firmicutes showed a negative correlation (r = −0.623), suggesting that the two may compete or complement each other in hydrogen metabolism. Desulfobacterota also showed a strong correlation with the “aromatic” signature (r = 0.629), suggesting that they may mediate the degradation of aromatic compounds. Genus-level analysis (Figure 4E) further revealed that Rikenellaceae_RC9_gut_group was significantly positively correlated with DHA content (r = 0.664), while Alistipes was strongly negatively correlated with the “arom.aliph” signature (r = −0.832), suggesting that they may be involved in lipid metabolism and the regulation of aromatic-aliphatic flavor compounds, respectively.

In addition, significant interactions between microbial communities and phenotypic indicators were observed. At the phylum level (Figure 4C), the leg muscle electronic nose “broad.methane” signal was strongly positively correlated with Spirochaetota (r = 0.699), while egg glycine content was negatively correlated with Proteobacteria (r = −0.360), potentially reflecting its regulatory role in methane production and amino acid metabolism. At the genus level (Figure 4D), Faecalibacterium was positively correlated with the egg electronic nose signal “arom.aliph” (r = 0.357). Rikenellaceae_RC9_gut_group was again significantly positively correlated with DHA content (r = 0.664), further supporting its role in regulating egg lipid composition and nutritional value.

## 4. Discussion

The decline in product quality during the late laying period represents a significant challenge to the sustainability of the poultry industry. This study demonstrates that dietary supplementation with BCEs systemically enhances the quality of eggs and meat from aged laying hens. BCEs are a botanical compound composed of crude extracts of various Chinese herbal medicines, including honeysuckle, mulberry leaves, *Codonopsis pilosula*, and magnolia bark. They are rich in polysaccharides, flavonoids, and chlorogenic acid. Different botanical ingredients exhibit synergy and complementarity in their chemical structures and targets and can coordinately regulate metabolic homeostasis through multi-target and multi-pathway mechanisms, demonstrating systemic advantages that are distinct from single botanical additives [13,14]. Existing studies have also confirmed that Chinese herbal compounds have great application potential in improving the health status and product quality of aged laying hens [15,16]. More importantly, our integrated analysis reveals that these improvements are mediated through a coordinated “gut microbiota-host metabolism” axis, underscoring the potential of BCEs as a novel functional feed strategy.

In terms of flavor quality, electronic nose testing showed that BCE treatment significantly enhanced the release of aromatic (arom-aliph) and fat-soluble (methane-aliph) volatiles in eggs, while reducing hydrogen signals related to off-flavors, indicating that it helps optimize the composition of flavor substances and reduce the accumulation of off-flavor substances [17]. Similar plant polyphenols, such as resveratrol, have been shown to interfere with the aroma metabolic pathway and optimize the volatile components of eggs [18]. In addition, chlorogenic acid in BCEs may indirectly affect the generation and transformation of volatile precursors by regulating liver lipid metabolism and antioxidant pathways [19]. This study found that the flavor improvement effect was particularly significant in cooked eggs, which may be related to the formula promoting the release of aroma precursors under thermal processing conditions or inhibiting oxidative degradation, which is consistent with the report of Zhang et al. that antioxidants improve the stability of egg aroma [20].

Fatty acid analysis revealed that BCEs significantly promoted the enrichment of high-nutritional-value n-3 polyunsaturated fatty acids (such as C22:6, DHA) in egg yolk without markedly increasing the level of saturated fatty acids (such as C18:0), while also simultaneously improving the n-6/n-3 ratio. This beneficial modulation may be achieved by promoting fatty acid elongation reactions (such as increasing C20:1 and decreasing C18:3n6), thereby improving lipid metabolism efficiency [21]. Related studies have shown that polyphenols in plant residues can enhance DHA deposition and improve antioxidant capacity [22]. The synergistic effect of plant polyphenols and oils also has a positive impact on the enrichment and stability of n-3 PUFA [23]. The combined effects of polysaccharides and polyphenols in the BCEs formula likely work synergistically in lipid synthesis [24], transport, and deposition [25], thereby achieving comprehensive regulation of fatty acid composition [26]. Amino acid analysis results showed that BCE treatment led to a significant decrease in the content of glycine, methionine, and histidine in the egg white and yolk mixture, and the total amount of 16 amino acids showed a downward trend. Although amino acids are important indicators of the nutritional value of eggs, their excessive accumulation may lead to an increase in bitterness or an increase in ammonia nitrogen load [27]. Traditional Chinese medicine formulas can regulate protein synthesis and amino acid homeostasis by regulating the expression of amino acid synthases, mTOR signaling pathways, and transmembrane transporters [28]. This nutritional reconfiguration establishes a functionally complementary relationship between DHA enrichment and moderated amino acid levels. The substantial elevation of DHA—a fatty acid with well-documented significance in human nutrition—enhances the functional lipid profile of eggs [29]. Simultaneously, the selective reduction in specific amino acids potentially improves sensory characteristics by minimizing bitterness precursors [30] while optimizing nitrogen metabolism [31]. Consequently, BCEs mediate a fundamental shift in nutritional architecture, prioritizing high-value lipid deposition while maintaining protein quality. This strategic reallocation of metabolic resources transforms eggs from basic nourishment to functionally optimized food, enhancing health-promoting properties without compromising essential nutritional value.

In terms of slaughter performance, BCE treatment significantly improved the slaughter rate on the 60th day of the experiment. Although there was no statistical difference in body weight, eviscerated weight, and abdominal fat weight, the overall indicators showed an upward trend, suggesting that it may promote the development of the body cavity and fat storage in the early stage of late egg production. The temporal specificity of these effects—evident at 60 days but attenuated by 100 days—corresponds to the established concept of a critical window in nutritional physiology, wherein the organism’s responsiveness to dietary interventions is temporally restricted to specific developmental or metabolic transition phases [32]. This stage-dependent efficacy indicates that BCEs primarily modulate physiological processes during periods of heightened metabolic plasticity, with diminished effects following the establishment of a new homeostatic equilibrium [33]. Further longitudinal investigations are required to precisely define the temporal boundaries of this responsive period and to clarify the underlying regulatory mechanisms governing this time-limited physiological response [34].

Plant active ingredients such as chlorogenic acid and quercetin are believed to regulate fatty acid synthase (FAS) and peroxisome proliferator-activated receptors (PPARs) pathways [35], and promote tissue lipid accumulation [36]. Muscle nutritional analysis showed that long-term feeding of BCEs significantly reduced the crude protein content of breast muscle and increased the crude fat level. The leg muscle also showed a similar trend, suggesting that it may induce protein-fat metabolic redistribution in muscle. This change may be related to the regulation of protein synthesis and lipid deposition by flavonoids in BCEs through the AMPK/mTOR pathway [37]; similar mechanisms have also been reported in mammalian studies [38]. This nutrient repartitioning phenomenon represents a beneficial improvement in meat quality rather than a nutritional deficiency. The reduction in crude protein, accompanied by increased intramuscular fat, reflects a strategic shift in energy substrate allocation that enhances the sensory characteristics of meat. For spent laying hens, which typically exhibit tough texture and poor palatability, this metabolic reprogramming significantly improves tenderness and flavor intensity [39]. Intramuscular fat deposition serves as a natural tenderizer by separating muscle fibers and contributing to juiciness, while also serving as a reservoir for flavor precursors [40]. This mechanism aligns with the established positive correlation between moderate intramuscular fat content and meat quality attributes in poultry. Therefore, the BCE-induced metabolic shift optimizes the eating quality of spent hen meat through targeted nutrient repartitioning, representing a valuable approach to enhancing the utilization of this protein resource.

Although the protein content is slightly reduced, the moderate increase in fat content helps to improve the flavor, tenderness, and juiciness of the meat [41], which has a positive significance for improving the edible quality of eliminated chicken. Analysis of flavor compounds showed that BCE treatment could regulate the composition of volatile compounds such as aromatics, alcohols/aldehydes, and sulfur-containing compounds in chicken. At 60 days of age, the response to alcohol/aldehyde and methane-aliph compounds increased, indicating that BCEs may improve muscle flavor by regulating lipid oxidation and the production of sulfur-containing volatiles [42]. At 100 days of age, some odor signals showed a reversal of response, suggesting that their regulation is metabolically adaptive and accumulation-dependent [43].

In terms of intestinal microecology, although the difference in the alpha diversity index did not reach a significant level, the species richness of the BCEs group showed an upward trend, suggesting that it may have a positive effect on microbial stability and ecological redundancy. The Venn diagram and species composition bar chart showed that BCE treatment significantly reshaped the structure of the cecal microbial community, and the abundance of some dominant OTUs changed. Plant active ingredients such as polysaccharides and polyphenols can serve as microbial substrates or regulatory factors, promoting the enrichment of beneficial bacteria and inhibiting potentially harmful bacteria [44]. In this study, BCEs significantly reduced the relative abundance of Slackia and Eubacterium genera, which are closely related to protein fermentation and endotoxin production [45], their reduction helps maintain the intestinal mucosal barrier and the stability of the inflammatory state [46]. It is particularly noteworthy that alpha diversity primarily reflects community richness and evenness, whereas functional differences often stem from the regulation of specific core bacterial genera. Our results demonstrate that although overall diversity showed no significant changes, key functional bacteria (such as *Slackia* and *Eubacterium*) were significantly affected, indicating that BCEs possess a selective microecological intervention capability. This finding aligns with the current perspective in microbial ecology: the specific regulation of functional bacterial groups is more decisive for host health than overall diversity changes [47,48].

Beta diversity analysis revealed significant separation between the BCEs group and the control group at the genus level, suggesting that BCEs primarily achieve microecological intervention by regulating low- and medium-abundance microbiota. PICRUSt2 functional prediction revealed that BCEs significantly enhanced functional pathways related to secondary metabolites (short-chain fatty acids, phenols, etc.), suggesting that they can enhance microbial metabolic activity and contribute to the maintenance of host energy metabolism and immune homeostasis.

Metabolomics results further support these conclusions. PCA and OPLS-DA analyses revealed that BCEs significantly altered the serum metabolic profile of laying hens in the late laying period. Among the differentially expressed metabolites, substances with antioxidant and lipid-regulating functions, such as niacin, apigenin, and paraxanthine, were significantly upregulated. KEGG analysis revealed that the differentially expressed metabolites were enriched in multiple metabolic and signaling pathways, including AMPK, calcium signaling, and caffeine metabolism, suggesting that BCEs may improve lipid metabolism and inflammation by activating the AMPK pathway, a hub of energy metabolism. Furthermore, adipose cytokine signaling, insulin resistance, and cholesterol metabolism pathways were also affected, further demonstrating that BCEs have the potential to regulate metabolic networks at multiple pathways and levels.

In the correlation between serum metabolites and phenotypic characteristics, paraxanthine showed a significant positive correlation with the “hydrogen” signal in the leg muscle electronic nose and a negative correlation with the amino acid glycine, suggesting that it may be involved in the regulation of muscle mitochondrial energy metabolism and amino acid synthesis/transport balance. Literature shows that paraxanthine has the potential to promote muscle fat oxidation and energy allocation [49]. In addition, nicotinuric acid showed a moderate negative correlation with methionine, indicating that it may indirectly participate in the regulation of nitrogen metabolism by intervening in the purine-sulfur amino acid pathway. Some serum metabolites may also affect the formation of flavor precursors. Bacteriopheophytin a was highly positively correlated with the “sulfur.organic” signal in eggs, which may be related to the accumulation or release of sulfur-containing flavor precursors. Sulfur-containing compounds are key components of egg aroma, and their release is regulated by multiple factors such as lipid oxidation, protein degradation, and the Maillard reaction [50]. This suggests that BCEs change the basis of flavor substances by regulating the metabolite spectrum.

In the interaction between microbiota, metabolites, and flavor nutrition, the intestinal microbiota plays a bridging role. Phylum-level analysis showed that Bacteroidota was significantly positively correlated with hydrogen flavor characteristics, while *Firmicutes* was negatively correlated, suggesting that the two may have a metabolic division of labor in the fermentation end products, hydrogen production, and utilization pathways, thereby affecting the smell of meat. In addition, Desulfobacterota was positively correlated with the “aromatic” signal, indicating that it may play a role in the degradation or transformation of aromatic compounds, affecting the flavor formation pathway [19]. At the genus level, Rikenellaceae_RC9_gut_group showed a significant positive correlation with the DHA content in egg yolk. This genus is believed to be related to the synthesis or accumulation of n-3 polyunsaturated fatty acids [51], and its functions may involve bile acid transport, lipid absorption regulation, and other links. In contrast, Alistipes showed a strong negative correlation with aromatic-aliphatic odor signals (arom.aliph). The activity of this bacterial community in amino acid degradation and short-chain fatty acid metabolism may lead to the degradation of flavor-active precursors [52], thereby reducing aroma complexity.

The microbial community is also directly related to flavor and nutritional indicators. At the phylum level, Spirochaetota was positively correlated with leg muscle methane signals, suggesting that it may be involved in methane metabolism or the production of lipid fermentation end products [53], and Proteobacteria was negatively correlated with glycine, possibly reflecting its participation in amino acid degradation [54]. At the genus level, Faecalibacterium was positively correlated with egg “arom.aliph” signals, while the positive correlation between Rikenellaceae_RC9_gut_group and DHA was again verified, emphasizing its central role in lipid nutrition improvement [55].

Overall, BCEs not only influence the composition of the gut microbiome but also achieve multi-dimensional regulation of flavor and nutrition through metabolite pathways. Their active ingredients, such as polysaccharides, flavonoids, and chlorogenic acid, complement each other in regulating fatty acid synthesis, amino acid homeostasis, and antioxidant pathways, establishing a systematic regulatory network centered on the “microbiome-metabolism-phenotype” network. This study acknowledges several limitations, including the absence of commercial additive comparisons, the 100-day duration potentially limiting long-term effect assessment, and incomplete safety evaluation data. Future research should prioritize comparative efficacy studies, extended trial periods, and systematic safety assessments to facilitate BCEs’ translation into practical applications.

## 5. Conclusions

This study demonstrated that BCEs exerted multidimensional improvements in laying hens during the late egg-laying period. BCEs enhanced the flavor and nutritional composition of eggs by promoting the deposition of n-3 polyunsaturated fatty acids, regulating amino acid metabolism, and reducing off-flavor precursors. In muscle, BCEs improved protein–lipid metabolic balance and flavor compound formation, thereby enhancing the quality of culled hens. Furthermore, BCEs modulated the intestinal microbiota by reducing harmful taxa and enriching functional bacteria, such as Rikenellaceae_RC9_gut_group, while promoting the biosynthesis of short-chain fatty acids and aromatic metabolites. Serum metabolomics revealed significant alterations in metabolic pathways related to energy, lipid, and antioxidant regulation, including AMPK and calcium signaling. Together, these findings suggest that BCEs regulate egg and meat quality through a microbiota–metabolome–phenotype network, providing a theoretical basis and practical reference for the development of functional feed strategies to improve the performance and product value of aged laying hens.

## Figures and Tables

**Table 1:** Basic diet composition and nutritional level.

Ingredients	Content, %	Nutrition Levels	Content, %
Corn	61.20	Metabolic energy (MJ/kg)	11.55
Soybean meal	24.00	Crude protein	16.10
Soybean oil	3.00	Calcium	3.67
Stone powder	9.00	Phosphorus	0.28
Calcium bicarbonate	1.00	Lysine	0.91
Salt	0.35	Methionine and cystine	0.72
Methionine	0.20	Threonine	0.75
Threonine	0.15		
Premix ^1^	1.10		
Total	100.00		

^1^ The premix provides vitamin A 10,000 IU, vitamin D3 3500 IU, vitamin E 25 mg, vitamin K3 3 mg, vitamin B 12.5 mg, vitamin B 26.5 mg, vitamin B 68.5 mg, vitamin B 120.03 mg, niacinamide 30 mg, calcium pantothenate 25 mg, folic acid 1.0 mg, biotin 0.2 mg, choline 0.7 g, phytase 0.1 g, manganese 80 mg, zinc 75 mg, copper 8.0 mg, iron 80 mg, iodine 0.8 mg, and selenium 0.3 mg per kilogram of diet; the nutritional levels are all calculated values.

**Table 2:** Effects of feeding BCEs on egg electronic nose.

Term	Aromatic-Hydrocarbons	Broadrange	Aromatic-Oxygenated	Hydrogen	Arom-Aliph
60 d raw egg					
CON	1.025 ^b^	1.035 ^b^	1.005 ^b^	1.009	1.005 ^b^
BCEs	1.037 ^a^	1.069 ^a^	1.007 ^a^	0.997	1.007 ^a^
60 d cooked egg					
CON	1.025 ^a^	1.051 ^a^	1.009 ^a^	0.852 ^b^	1.008 ^a^
BCEs	1.020 ^b^	1.033 ^b^	1.002 ^b^	0.993 ^a^	1.004 ^b^
100 d raw egg					
CON	1.033 ^a^	1.058	1.008 ^a^	0.997 ^b^	1.007 ^a^
BCEs	1.014 ^b^	1.051	1.002 ^b^	1.003 ^a^	1.001 ^b^
100 d cooked egg					
CON	1.016 ^a^	1.001 ^b^	1.005 ^a^	0.998 ^b^	1.003
BCEs	1.006 ^b^	1.007 ^a^	1.002 ^b^	1.005 ^a^	1.001

Different superscript letters indicate significant differences (*p* < 0.05), while the same letters indicate no significant difference (*p* > 0.05).

**Table 3:** Effects of feeding BCEs on egg electronic nose.

Term	Broad-Methane	Sulfur-Organic	Broad-Alcohol	Sulph-Chlor	Methane-Aliph
60 d raw egg					
CON	0.967	0.846 ^b^	0.987	0.903 ^a^	1.004 ^a^
BCEs	0.941	0.866 ^a^	0.948	0.825 ^b^	0.991 ^b^
60 d cooked egg					
CON	0.971 ^b^	0.768 ^b^	0.973	0.778 ^a^	1.002 ^a^
BCEs	0.983 ^a^	0.785 ^a^	0.969	0.697 ^b^	1.000 ^b^
100 d raw egg					
CON	0.968 ^b^	0.929	0.879	0.878 ^b^	0.992 ^b^
BCEs	0.993 ^a^	0.929	0.926	0.960 ^a^	1.003 ^a^
100 d cooked egg					
CON	0.960 ^b^	0.915	0.988	0.947	0.998 ^b^
BCEs	1.003 ^a^	0.914	0.993	0.943	1.002 ^a^

Different superscript letters indicate significant differences (*p* < 0.05), while the same letters indicate no significant difference (*p* > 0.05).

**Table 4:** Effects of feeding BCEs on egg yolk fatty acids.

Term	C14:0	C14:1n5	C15:0	C16:0	C16:1n7	C17:0	C17:1n7	C18:0
60 d								
CON	0.017	0.003	0.003	1.266	0.144	0.008	0.006	0.451
BCEs	0.02	0.004	0.003	1.505	0.166	0.009	0.006	0.533
100 d								
CON	0.012	0.002	0.002	0.852	0.089	0.005	0.003 ^b^	0.304
BCEs	0.013	0.002	0.002	0.89	0.094	0.006	0.004 ^a^	0.332

Myristic acid: C14:0, Myristoleic acid: C14:1n5, Pentadecanoic acid: C15:0, Palmitic acid: C16:0, Palmitoleic acid: C16:1n7, Heptadecanoic acid: C17:0, cis-10-Heptadecenoic acid: C17:1n7, Stearic acid: C18:0. Different superscript letters indicate significant differences (*p* < 0.05), while the same letters indicate no significant difference (*p* > 0.05).

**Table 5:** Effects of feeding BCEs on egg yolk fatty acids.

Term	C18:1n9c	C18:2n6c	C18:3n3	C20:1	C20:2	C20:3n6	C22:1n9	C20:4n6	C22:6n3
60 d									
CON	2.152	0.961	0.044	0.012	0.013	0.009	0.007	0.087	0.038
BCEs	2.561	1.105	0.048	0.014	0.015	0.01	0.004	0.101	0.04
100 d									
CON	1.416	0.668	0.028 ^b^	0.008	0.008	0.006	0.006 ^a^	0.068	0.029
BCEs	1.613	0.722	0.033 ^a^	0.009	0.009	0.006	0.004 ^b^	0.065	0.031

Oleic acid (cis-9-octadecenoic acid): C18:1n9c, Linoleic acid (cis-9,12-octadecadienoic acid): C18:2n6c, Alpha-linolenic acid: C18:3n3, C20:1 refers to C20:1n9 (cis-11-eicosenoic acid), and C20:2 refers to C20:2n-6 (all-cis-11,14-eicosadienoic acidC, Dihomo-γ-linolenic acid: C20:3n6, cis-13-docosenoic acid: C22:1n9, Arachidonic acid: C20:4n6, Docosahexaenoic acid: C22:6n3. Different superscript letters indicate significant differences (*p* < 0.05), while the same letters indicate no significant difference (*p* > 0.05).

**Table 6:** Effects of feeding BCEs on amino acids in egg yolk and egg white mixture.

Term	Asp	Thr	Ser	Glu	Gly	Ala	Val	Met
60 d								
CON	1.24 ^a^	0.595 ^a^	0.859 ^a^	1.653 ^a^	0.411 ^a^	0.7 ^a^	0.758 ^a^	0.406 ^a^
BCEs	1.196 ^b^	0.564 ^b^	0.826 ^b^	1.549 ^b^	0.396 ^b^	0.674 ^b^	0.733 ^b^	0.388 ^b^
100 d								
CON	1.214 ^a^	0.581	0.852 ^a^	1.629 ^a^	0.407 ^a^	0.686 ^a^	0.735 ^a^	0.41 ^a^
BCEs	1.161 ^b^	0.562	0.82 ^b^	1.547 ^b^	0.386 ^b^	0.656 ^b^	0.693 ^b^	0.382 ^b^

Aspartic acid: Asp, Threonine: Thr, Serine: Ser, Glutamic acid: Glu, Glycine: Gly, Alanine: Ala, Valine: Val, Methionine: Met. Different superscript letters indicate significant differences (*p* < 0.05), while the same letters indicate no significant difference (*p* > 0.05).

**Table 7:** Effects of feeding BCEs on amino acids in egg yolk and egg white mixture.

Term	Ile	Leu	Tyr	Phe	Lys	His	Arg	Pro	Total Amino Acids
60 d									
CON	0.618	1.018	0.536	0.662 ^a^	0.893	0.304 ^a^	0.797 ^a^	0.477 ^a^	11.929 ^a^
BCEs	0.602	1.006	0.543	0.637 ^b^	0.907	0.286 ^b^	0.757 ^b^	0.443 ^b^	11.503 ^b^
100 d									
CON	0.59 ^a^	0.981 ^a^	0.507	0.659 ^a^	0.859 ^a^	0.29 ^a^	0.766	0.467 ^a^	11.622 ^a^
BCEs	0.555 ^b^	0.929 ^b^	0.487	0.62 ^b^	0.825 ^b^	0.276 ^b^	0.741	0.448 ^b^	11.094 ^b^

Isoleucine: Ile, Leucine: Leu, Tyrosine: Tyr, Phenylalanine: Phe, Lysine: Lys, Histidine: His, Arginine: Arg, Proline: Pro. Different superscript letters indicate significant differences (*p* < 0.05), while the same letters indicate no significant difference (*p* > 0.05).

**Table 8:** Effects of feeding BCEs on slaughter performance of laying hens in the late laying period.

Term	Pre-Slaughter Live Weight, kg	Carcass Weight, kg	Full Eviscerated Weight, kg	Semi-Eviscerated Weight, kg	Abdominal Fat Weight, g	Pectoral Weight, kg	Leg Muscle Weight, kg
60 d							
CON	2.083	1.886	1.294	1.557	91	0.195	0.232
BCEs	2.16	2.004	1.346	1.626	107.38	0.221	0.232
100 d	Pre-slaughter live weight, kg	Carcass weight, kg	Full eviscerate weight, kg	Semi-eviscerated weight, kg	Abdominal fat weight, g	Pectoral weight, kg	Leg muscle weight, kg
CON	2.02	1.867	1.263	1.568	77.002	0.175	0.234
BCEs	2.047	1.924	1.302	1.605	103.314	0.178	0.212
60 d	Slaughter percentag, %	Semi-eviscerated rate, %	Eviscerated rate, %	Pectoral rate, %	Leg muscle rate, %	Abdominal fat rate, %	Lean rate, %
CON	0.905 ^b^	0.747	0.621	0.15	0.179	0.065	0.329
BCEs	0.928 ^a^	0.753	0.623	0.154	0.173	0.073	0.327
100 d	Slaughter percentag, %	Semi-eviscerated rate, %	Eviscerated rate, %	Pectoral rate, %	Leg muscle rate, %	Abdominal fat rate, %	Lean rate, %
CON	0.924	0.776	0.626	0.138	0.186	0.057	0.324
BCEs	0.941	0.784	0.634	0.137	0.166	0.072	0.303

Different superscript letters indicate significant differences (*p* < 0.05), while the same letters indicate no significant difference (*p* > 0.05).

**Table 9:** Effects of feeding BCEs on meat quality of laying hens in the late laying period.

Pectoral	pH	Meat Color-L*	Meat Color-a*	Meat Color-b*	Drip Loss Rate, %
60 d					
CON	5.844	55.495	5.135	9.445	2.067
BCEs	5.953	57.087	3.672	9.988	2.497
100 d					
CON	5.96	51.738	5.689	8.508	4.567
BCEs	5.751	51.414	5.292	8.283	4.873

**Table 10:** Effects of feeding BCEs on meat quality of laying hens in the late laying period.

Leg Muscle	pH	Meat Color-L*	Meat Color-a*	Meat Color-b*	Drip Loss Rate, %
60 d					
CON	6.022	46.451	14.756	10.166	6.373
BCEs	5.96	46.397	14.803	7.891	5.608
100 d					
CON	6.022	46.451	14.756	10.166	6.373
BCEs	5.96	46.397	14.803	7.891	5.608

**Table 11:** Effects of feeding BCEs on the nutritional components of laying hen meat in the late laying period.

	Pectoral		Leg Muscle	
Term	Crude Protein	Crude Fat	Crude Protein	Crude Fat
60 d				
CON	0.874	0.065	0.724	0.195
BCEs	0.888	0.057	0.515	0.204
100 d				
CON	0.882 ^a^	0.050 ^b^	0.79	0.13
BCEs	0.861 ^b^	0.068 ^a^	0.668	0.256

Different superscript letters indicate significant differences (*p* < 0.05), while the same letters indicate no significant difference (*p* > 0.05).

**Table 12:** Effects of feeding BCEs on laying hen meat electronic nose in late laying period.

Term	Aromatic-Hydrocarbons	Broadrange	Aromatic-Oxygenated	Hydrogen	Arom-Aliph	Broad-Methane	Sulfur-Organic	Broad-Alcohol	Sulph-Chlor	Methane-Aliph
60 d Pectoral										
CON	1.041 ^a^	1.085 ^a^	1.010 ^a^	0.997	1.011 ^a^	0.930 ^b^	0.834 ^a^	0.910 ^b^	0.888 ^a^	0.993 ^a^
BCEs	1.013 ^b^	0.987 ^b^	1.002 ^b^	1	1.003 ^b^	0.975 ^a^	0.802 ^b^	1.195 ^a^	0.846 ^b^	0.983 ^b^
60 d Leg muscle										
CON	1.021	1.034	1.005	1.000 ^b^	1.005 ^b^	0.968 ^b^	0.833 ^a^	1.003 ^b^	0.867 ^a^	1.005
BCEs	1.021	1.026	1.007	1.002 ^a^	1.009 ^a^	0.990 ^a^	0.786 ^b^	1.114 ^a^	0.825 ^b^	1.001
100 d Pectoral										
CON	1.012 ^b^	0.988 ^b^	1.002	0.999 ^b^	1.002 ^b^	0.98	0.798 ^b^	1.172 ^a^	0.845 ^b^	0.999 ^b^
BCEs	1.035 ^a^	1.079 ^a^	1.005	1.008 ^a^	1.005 ^a^	0.967	0.891 ^a^	0.965 ^b^	0.932 ^a^	1.037 ^a^
100 d Leg muscle										
CON	1.014 ^b^	0.996 ^b^	1.004	0.994 ^b^	1.005	0.965	0.778 ^b^	0.901 ^b^	0.821 ^b^	0.996 ^b^
BCEs	1.026 ^a^	1.045 ^a^	1.005	1.004 ^a^	1.005	0.975	0.830 ^a^	1.002 ^a^	0.877 ^a^	1.003 ^a^

Different superscript letters indicate significant differences (*p* < 0.05), while the same letters indicate no significant difference (*p* > 0.05).

## Data Availability

The original contributions presented in this study are included in the article/Supplementary Materials. Further inquiries can be directed to the corresponding author.

## Supplementary Materials

The following supporting information can be downloaded at: https://www.mdpi.com/article/10.3390/foods14203480/s1, Figure S1: Alpha diversity analysis; Figure S2: Venn diagrams of species, Bar chart analysis of community composition; Figure S3: β-diversity analysis; Table S1: Electronic nose sensor names and performance descriptions; Table S2: Effects of feeding BCEs on egg electronic nose; Table S3: Effects of feeding BCEs on egg electronic nose; Table S4: Effects of feeding BCEs on egg yolk fatty acids; Table S5: Effects of feeding BCEs on egg yolk fatty acids; Table S6: Effects of feeding BCEs on amino acids in egg yolk and egg white mixture; Table S7: Effects of feeding BCEs on amino acids in egg yolk and egg white mixture; Table S8: Effects of feeding BCEs on slaughter performance of laying hens in the late laying period; Table S9: Effects of feeding BCEs on meat quality of laying hens in the late laying period; Table S10: Effects of feeding BCEs on meat quality of laying hens in the late laying period; Table S11: Effects of feeding BCEs on the nutritional components of laying hen meat in the late laying period; Table S12: Effects of feeding BCEs on laying hen meat electronic nose in late laying period.

## Abbreviations

The following abbreviations are used in this manuscript:

BCEs	Botanical Crude Extracts
n-3 PUFAs	Omega-3 Polyunsaturated Fatty Acids
n-6/n-3	Ratio of Omega-6 to Omega-3 Fatty Acids
DHA	Docosahexaenoic Acid
AMPK	AMP-Activated Protein Kinase
mTOR	Mechanistic Target of Rapamycin
FAS	Fatty Acid Synthase
PPARs	Peroxisome Proliferator-Activated Receptors
OTUs	Operational Taxonomic Units
OPLS-DA	Orthogonal Partial Least Squares Discriminant Analysis
KEGG	Kyoto Encyclopedia of Genes and Genomes
PICRUSt2	Phylogenetic Investigation of Communities by Reconstruction of Unobserved States
HPLC	High-Performance Liquid Chromatography
FAP	Fatty Acid Profile
PCA	Principal Component Analysis
PUFA	Polyunsaturated Fatty Acid
SCFA	Short-Chain Fatty Acids
