# Supplementary figures and images for: Multi-Component Botanical Crude Extracts Improve Egg and Meat Quality in Late-Laying Hens Through Gut Microbiota Modulation
Source: Foods. 2025 Oct 12;14(20):3480. doi: 10.3390/foods14203480 (PMC12562306; doi:10.3390/foods14203480)

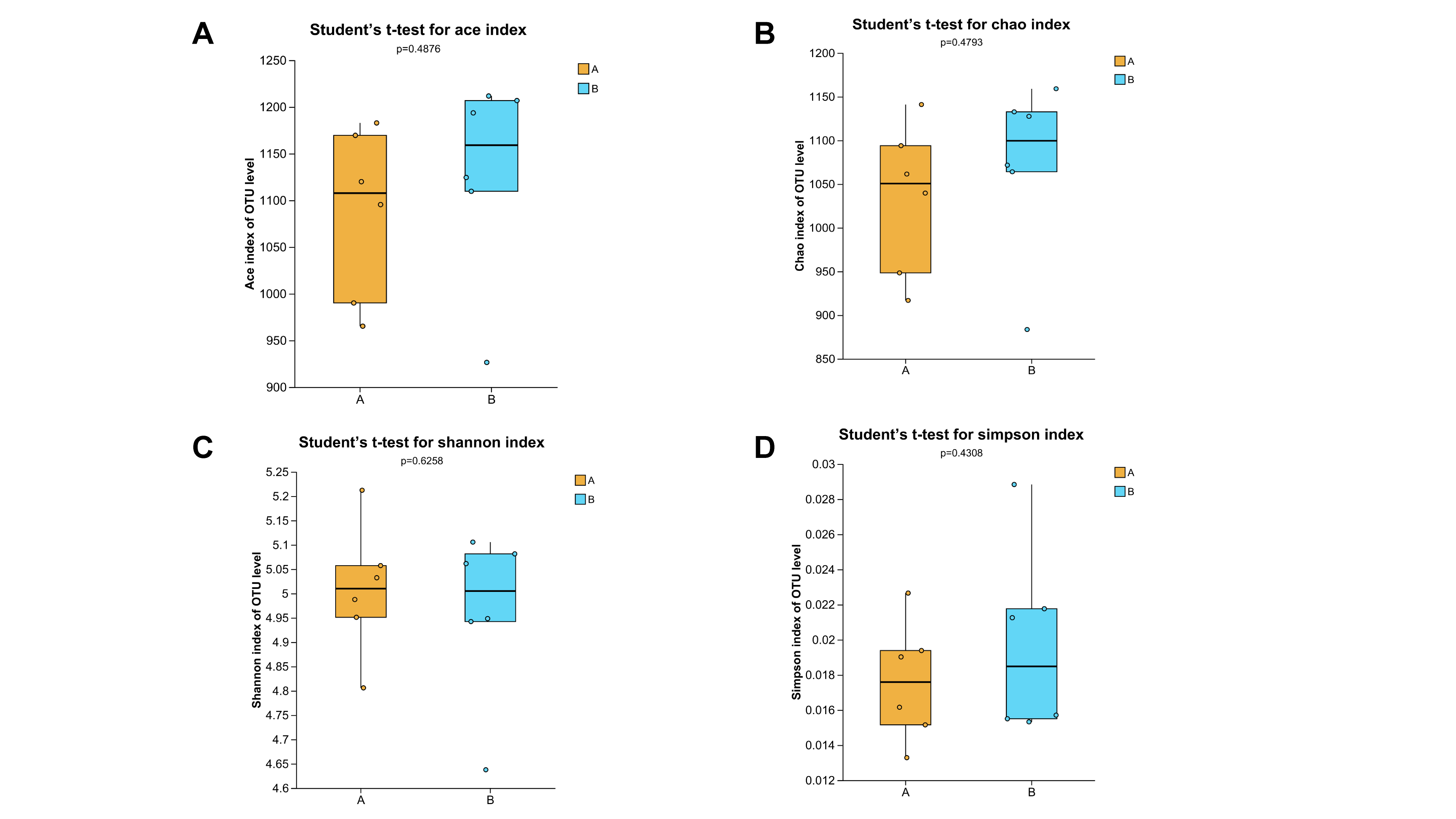

Supplement: Supplementary file 1 [file foods-14-03480-s001.zip › Figure S1.tif]

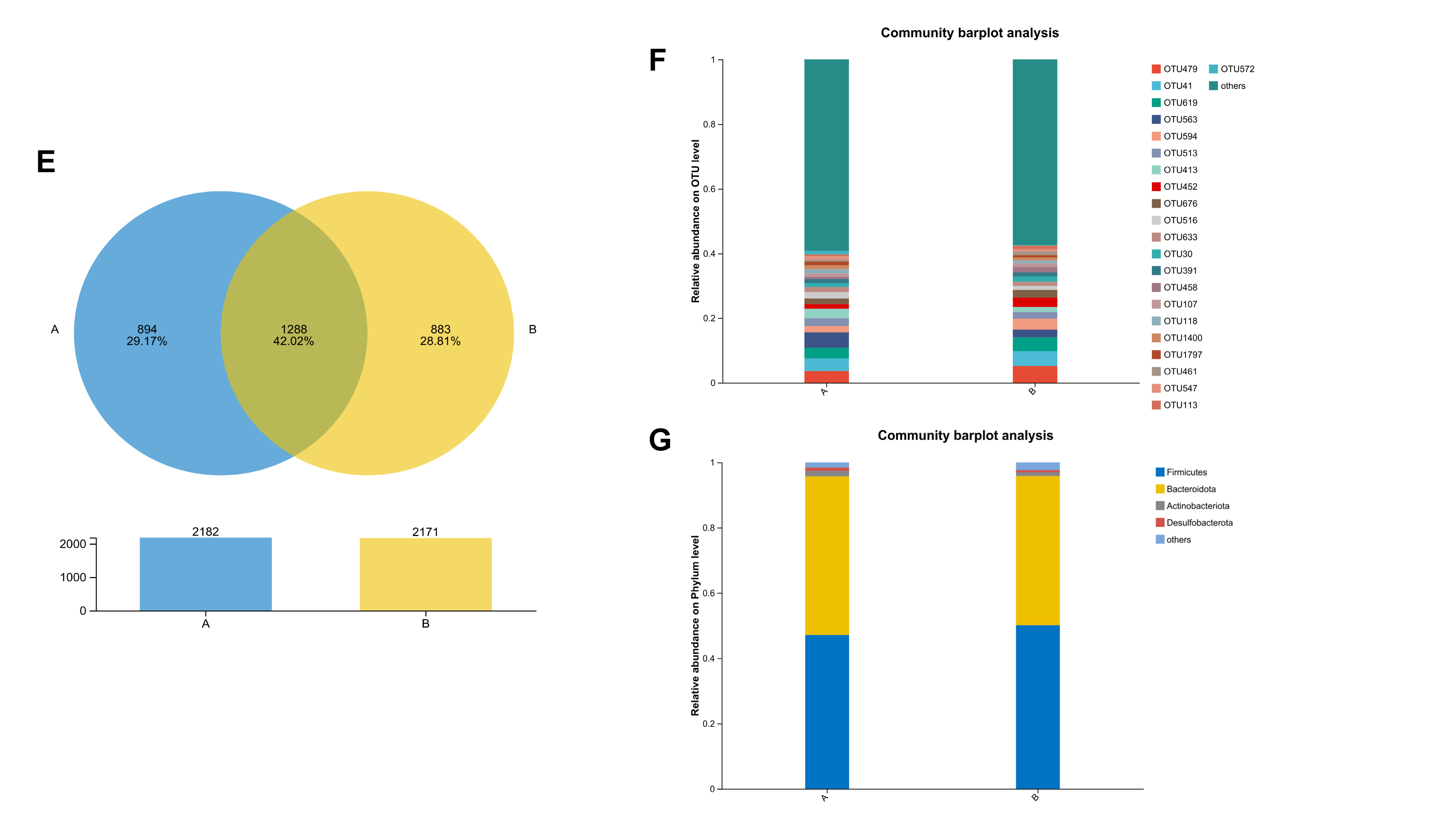

Supplement: Supplementary file 1 [file foods-14-03480-s001.zip › Figure S2.tif]

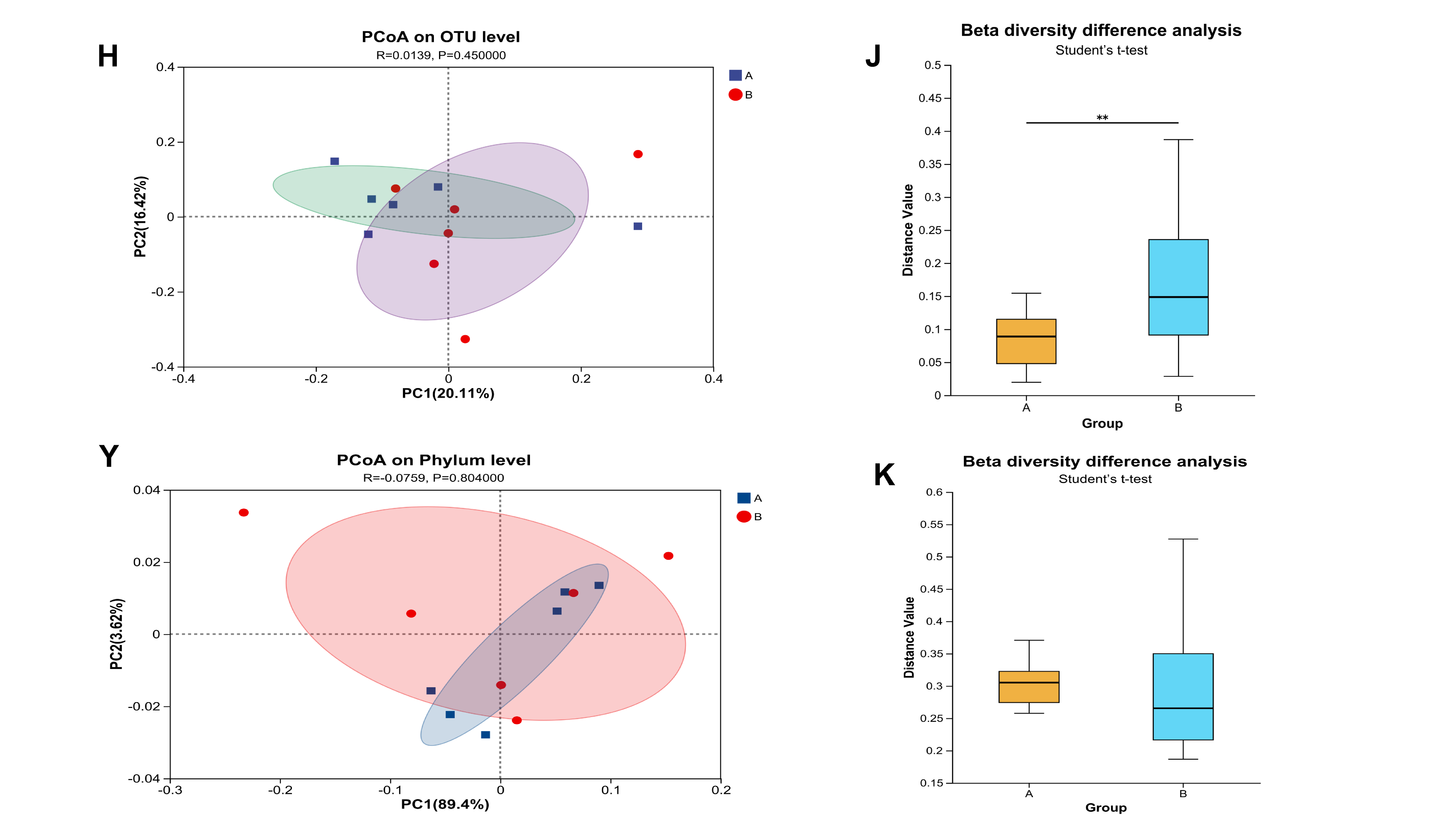

Supplement: Supplementary file 1 [file foods-14-03480-s001.zip › Figure S3.tif]
